# Effects of Simvastatin on Gene Expression and Alkaline Phosphatase Activity in the MG-63 Cell Line Treated With Hyperglycemia for Bone Regeneration

**DOI:** 10.7759/cureus.55482

**Published:** 2024-03-04

**Authors:** Athiban Inbarajan, Mubeena S, Alan Mathew Punnoose, Giri GVV, Anusha D, Jasline David J

**Affiliations:** 1 Prosthodontics, Sri Ramachandra Institute of Higher Education and Research, Chennai, IND; 2 Stem Cell and Regenerative Biology Laboratory, Sri Ramachandra Institute of Higher Education and Research, Chennai, IND; 3 Oral Surgery, Sri Ramachandra Institute of Higher Education and Research, Chennai, IND; 4 Pharmacology, Sri Ramachandra Institute of Higher Education and Research, Chennai, IND; 5 Oral Maxillofacial Surgery, Madha Dental College, Chennai, IND

**Keywords:** simvastatin, osteogenic differentiation, mg-63 cell lines, implants, hyperglycemia, antiresorptive drugs

## Abstract

Background

Dental implants have become a widespread treatment option for replacing missing teeth. Adequate bone is required for the placement of dental implants, in the absence of which, augmentation by bone regeneration is done. Antiresorptive drugs are used as treatment procedures for bone regeneration. One such antiresorptive drug is simvastatin (SV), a 3-hydroxy-3-methylglutaryl coenzyme used to manage hyperlipidemia. It reduces serum cholesterol levels and has an advantageous effect on new bone formation. Various studies establish that SV stimulates bone morphogenetic protein (BMP)-2 expression and leads to bone formation. SV prevents the production of isoprenoids and mevalonate, which are essential for osteoclastogenesis and contribute to the bone-sparing effect.

Aim

The aim of the study was to investigate the osteoregenerative activity of SV in the osteoblast-like cell models, MG-63 cell line, with hyperglycemic conditions.

Methodology

MG-63 cultures were established under high glucose concentrations during the experiments and cultured with SV concentrations of 1 µM and 3 µM. The quantification of the expression of the genes, namely, BMP-2 and osteocalcin (OCN) was done by real-time quantitative polymerase chain reaction (RTqPCR). The measurement of alkaline phosphatase activity in the SV-treated cells was also determined.

Results

According to the results of the study, SV had osteoprotective properties resulting from the inhibition of osteoclast stimulation and osteoinductive properties, facilitated by BMP-2 and OCN. In addition, SV at concentrations of 1 µM and 3 µM increased the gene expression of BMP-2 and OCN in the MG-63 cell line.

Conclusion

The results of this study demonstrated that SV had a significant and direct effect on osteogenesis in osteoblasts in vitro.

## Introduction

The dental implant treatment option is becoming a widespread treatment for replacing missing teeth in edentulous patients. Most clinical studies indicate that diabetes is no contraindication for implant placement on the condition that it is under metabolic control [1]. The bone consists of organic and inorganic materials, as well as cells that synthesize, remodel, and conserve bone. The bone cells that are made of osteocytes are surrounded by the bone matrix, sensing pressure to initiate bone resorption by osteoclasts [2,3]. Simvastatin (SV), which is a 3-hydroxy-3-methyglutaryl coenzyme A (HMG-CoA) competitive inhibitor, is used for reducing cholesterol and reducing the risk of a heart attack [4]. SV has been used for the treatment of hypercholesterolemia, although statins were initially reported to stimulate rodent bone formation in association with increased expression levels of bone morphogenetic protein (BMP)-2 in bone cells [5]. SV reduces the risk of fracture in elderly and osteoporotic patients and increases bone mineral density in humans. Statins fall into one of two categories: hydrophilic statins like pravastatin or hydrophobic statins like SV, atorvastatin, or cerivastatin [6]. Ayukawa et al. (2004) reported that the administration of SV increases the value of both the bone contact ratio and bone density [7]. This drug has the potential to improve the nature of osseointegration [7]. Chang et al. (2014) reported that biocompatible tantalum carbide (TaC) and TaC/amorphous carbon (a-C) coatings exhibited better cell viability of human osteosarcoma cell line MG-63 than the uncoated Ti and Ta-coated samples. Hence, the MG-63 cells used in the study were highly beneficial in improving in vitro biocompatibility [8]. Researchers have gained attention to the role of SV to induce bone morphogenic protein-2 (BMP-2) which is an important stimulator of osteoblastic differentiation. In various studies done both in humans and animals, SV was found to improve the process of fracture healing and heal bony defects. In the present study, evaluation of the cytotoxicity and differentiation under hyperglycemia of the SV-treated MG-63 cell line was done using real-time quantitative polymerase chain reaction (RTqPCR). The alkaline phosphatase (ALP) assay for the cells treated with SV was also done to measure and support that SV can inhibit osteoclast stimulation.

## Materials and methods

Cytotoxicity by MTT assay

Human osteosarcoma cells, MG-63, were seeded at a density of 2 × 10^5^ cells/well in 96-well plates. After the rapid growth of the cells at a temperature of 37 °C, the cultures were sustained for 72 hours in a medium comprising SV at concentrations ranging from 1μM to 1mM. The cytotoxicity was evaluated by 3-( 4,5-dimethylthiazol-2-yl)-2,5-diphenyltetrazolium bromide (MTT) assay by adding 10 μL MTT solution (5 mg/mL) to 100 μL of cell culture medium of each well and cultures were continued for 4 hours at a temperature of 37°C. The culture medium was then removed, and the resulting formazan crystals were dissolved in 100 μL of DMSO. The contents were then measured at a wavelength of 490 nm and the colorimetric variations were calculated using the microplate reader. The MTT assay was carried out in triplicate. The results were calculated using the optical density (OD) (OD treated cells/OD control [untreated] cells) ×100 to give the cell viability percentage.

Cell culture

The MG-63 cells were seeded in a flask at a density of 2 × 10^5^ cells/cm^2^ and incubated in DMEM medium added with 10% fetal bovine serum (FBS), L-glutamine (2 mM), 10 mM β-glycerophosphate, and 50 ng/ml L-ascorbic at 37 °C in 5% CO_2_ and 95% humidity. Once the cells reached 80% confluency, the cells were collected by trypsinization and were extensively cleaned with 10 mM phosphate-buffered saline (PBS), at a pH of 7.2. The cells were made into six groups at the termination of the second passage, as follows, Group A being the negative control (NC) containing medium only; Group B, the second group was the positive control (PC), the hyperglycemia group (HG) (35mM), Group C cells was induced with 10^−8^ M, dexamethasone, osteoblast differentiation medium (ODM), Group D was treated with hyperglycemia (HG) (35mM), with 10^−8^ M dexamethasone (ODM), Group E was the experimental group with 1 µM SV induced with hyperglycemia and the ODM, Group F was the experimental group with 3 µM SV induced with hyperglycemia and the ODM respectively. After three weeks of culture, ALP assay and real-time quantitative polymerase chain reaction (RTqPCR) were performed.

Total RNA isolation and RTqPCR study

The cells were then seeded at a density of 2 × 10^5^ cells/well in a 96-well plate. After the rapid growth of the cells, SV was added at concentrations of 1 µM and 3 µM and cultured. The total RNA was isolated from the cells using TRIzol® reagent, which is a monophasic solution of phenol and guanidinium isothiocyanate that simultaneously solubilizes biological material and denatures protein [9]. Lysates were extracted with chloroform and total RNA was precipitated with isopropanol. The experiment was done for three cultures. The amount and the value of the isolated RNA were studied using the Labman UV-VIS Spectrometer. A reaction mixture containing reverse transcriptase (MMLV) was used to transform 1.5 µg of total RNA into cDNA. For 10 minutes, cDNA was synthesized at 25 ℃. The template for the target genes was the cDNA. The SYBR® Green JumpStartTM Taq Ready MixTM was used for RTqPCR to measure the expression levels of the selected genes. The three primer sets used in the study were the following, 1. β - actin (F- 5′- AGCCATGTACGTAGCCATCC -3' / R- 5′- CTCTCAGCTGTGGTGGTGAA -3'), 2. 3OCN (F- 5’- GAAGCCCAGCGGTGCA -3’ / R- 5’- CACTACCTCGCTGCCCTCC -3’),3. BMP-2 (F- 5’- CGCCTCAAATCCAGCTGTAAG - 3’ / R- 5’- GGGCCACAATCCAGTCGTT-3’). Then the 2(-Delta Delta C(T)) method (∆∆Ct) method was used to measure the relative difference in values using Ct values of the samples, and relative changes in Ct values of the genes of interest. The final values were used to calculate the fold change of the target genes.

ALP assay

The cells were plated at a concentration of 2 × 10^5^ cells/well in 96 well plates. After the rapid growth of the cells, SV was added at two concentrations of 1 µM and 3 µM and then cultured. To measure the ALP activity in the samples, a pNPP alkaline phosphatase assay kit (Sigma, USA) was used. The colorimetric variations were measured using a spectrophotometer at 405 nm and the enzymes from the cells were quantified by comparing them using a typical value. The results of the ALP were then standardized to cell quantity at the completion of the experiments. ALP assay was performed in triplicate.

Statistical analysis

The analysis software IBM SPSS Statistics for Windows, Version 16 (Released 2007; IBM Corp., Armonk, New York, United States) was used for the statistical analysis. An independent t-test was used to compare the groups (simvastatin compound-treated versus control). The significance level was set at <0.05 for p. For each gene in the experimental groups, mean values and standard deviations were calculated [10]. The experimental hyperglycemic model investigated if SV can inhibit osteoclastic activity and promote osteoblastic activity. The current study's SV concentrations, 1 μM and 3 μM, were chosen based on a cytotoxicity study from the literature [11] and correlated with those used in previous human cell studies (0.001 μM to 10 μM) [12].

## Results

Cytotoxicity assay

**Figure 1:**
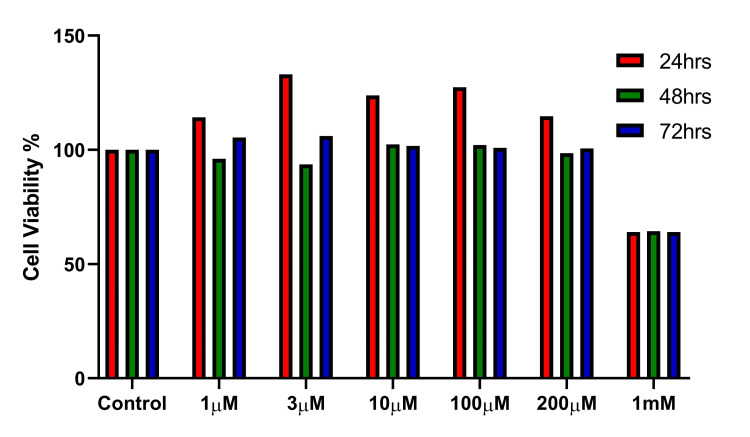
Cytotoxicity evaluation by MTT assay for different concentrations of simvastatin showing no toxicity for lower doses of 1 μM and 3 μM up to 72-hour time point MTT assay: MTT (3-[4,5-dimethylthiazol-2-yl]-2,5 diphenyl tetrazolium bromide) assay; mM: millimole; μM: micromole

The cytotoxicity evaluation was performed for a range of SV concentrations under normal culture conditions (Figure 1). The micromolar (μM) range from 1 to 200 of the SV had no toxic effect on the cell viability of the MG-63 cells used in the study. The SV exposure up to 72 hours was studied, revealing that concentrations from 1 μM to 200 μM had more than 95% cell viability for all time points. The SV concentration of 1mM showed more than a 35% decline in cell viability. Since the lower concentrations of 1 μM and 3 μM were already used in previously published literature we chose to proceed with the same for our further evaluations of osteogenic differentiation under hyperglycemia conditions.

Alkaline phosphatase assay

The outcome of the assay revealed that the ALP levels of groups treated with SV were lower than the negative control groups which contained the cell culture media only, but subsequently increased above the negative control level. There was a significant increase in alkaline phosphatase activity in hyperglycemic conditions at concentrations of 1 μM and 3 μM statin group (18% and 24% increase, respectively; p < 0.05*), compared with the negative control group. It has been reported that SV inhibits osteoclastic activity while promoting osteoblastic activity [13]. The control group and the 3 μM SV-treated group had the most increase in values (Table 1).

**Table 1:** ALP activity (units/ml) SV: Simvastatin; HG: hyperglycemia; mM: millimole; μM: micromole; ALP: alkaline phosphatase p < 0.05*: Statistically significant

S. No	Test samples	ALP Units/ml (in triplicate)			ALP Units/ml
1	Control	3.496	3.067	3.254	3.27233
2	Hyperglycemia (HG) (35mM)	0.1046	0.1222	0.128	0.11827
3	1 µM of simvastatin (SV)	0.1732	0.1812	0.1894	0.18127
4	3 µM of simvastatin (SV)	0.24414	0.25929	0.2624	0.25528

Results of RTqPCR gene expressions of BMP-2 and OCN analysis

**Figure 2:**
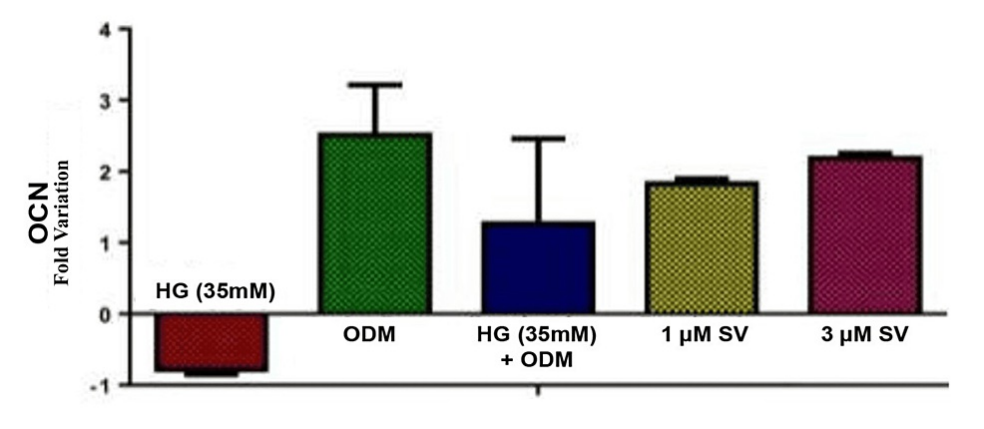
Osteocalcin gene expression MG-63 cell cultures undergoing osteogenic differentiation in hyperglycemic conditions treated with simvastatin p-value<0.05*: Statistically significant HG: Hyperglycemia; ODM: osteoblast differentiation media; SV: simvastatin; mM - millimole; μM: micromole; OCN: osteocalcin

**Figure 3:**
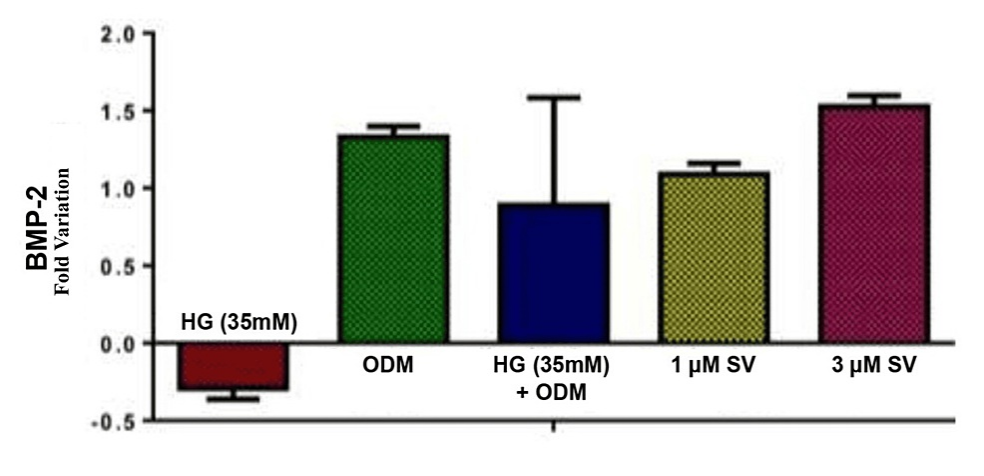
Bone morphogenetic protein-2 gene expression MG-63 cell cultures undergoing osteogenic differentiation in hyperglycemic conditions treated with simvastatin p-value<0.05*: Statistically significant HG: Hyperglycemia; ODM: osteoblast differentiation media; SV: simvastatin; mM - millimole; BMP: bone morphogenetic protein

Osteocalcin (OCN) gene expression was compared with the control group and the SV-treated groups (n=5) using an independent t-test. It was used at a significant level of 0.05. Gene expression levels differed significantly between the SV treatment groups and the control group. Figure 2 depicts a graphical representation of the mean expression fold change of OCN gene and Figure 3 depicts the graphical representation of the mean expression fold change of BMP-2 gene.

Based on the results, the expression level of the BMP-2 and OCN genes varied between the SV treatment groups and the control. The MG-63 cells treated with 1 µm and 3 µm of SV compound during osteogenic differentiation under hyperglycemic conditions showed an increase in fold change and upregulation of BMP-2 and OCN genes.

## Discussion

Chronic hyperglycemia is a hallmark of type II diabetes mellitus, a metabolic condition that also causes diabetic retinopathy and bone disease. The mechanisms that are thought to be responsible for the changes in bone that are brought on by type II diabetes mellitus have been the subject of debate for several years, and they are still unclear because other factors at play cover up changes in bone mineral density [14]. Increased expression of BMP-2 through activation of the gene promoter is linked to the enhancing effects of statins on bone formation [15]. By depleting mevalonate, the statins, compactin, and pitavastatin increase the gene expression for BMP-2 and OCN and promote the differentiation of embryonic stem cells into osteoblasts. According to Ohnaka et al., statins induce osteoblastic maturation, which is mediated by BMP-2 [16]. On the other hand, the SV-treated and control groups had significantly different levels of OCN expression, indicating that gene expression levels varied. The most abundant non-collagenous protein in bone is OCN, which is a protein that only osteoblasts secrete. In this study, gene expression analysis revealed that samples treated with SV expressed higher levels of OCN. Silvia Ruiz-Gaspa et al. reported a similar outcome and assessed the effect of SV and atorvastatin on osteoblast activity by analyzing cell proliferation, as well as collagen, OCN, and BMP-2 gene expression in primary human osteoblast (hOB) and MG-63 cultures [12]. In the drug-treated group, the expression of OCN was higher. This could indicate that mineralization is occurring more frequently than bone remodeling in the treated group. In contrast, more bone remodeling than mineralization occurs in the control group. The findings of the study showed that statins probably work through the BMP 2 pathway to form bones. In MC3T3-E1 murine osteoblastic cells, the hydrophobic statins SV, atorvastatin, and cerivastatin increase VEGF mRNA and protein expression [17]. The effects of various groups of statins on various cell lines directed the investigators to the conclusion that statins boost bone mineralization and proliferation [18]. In this study, the MG-63 cell line was chosen due to its stable characteristics in a wide range of cell culture passages and considered an appropriate in vitro model to analyze the biocompatibility and functionality of various materials [19]. Furthermore, it is very important to select the suitable glucose concentration in the culture medium according to individual cell types and experimental purposes to get the correct experimental results, especially for diabetes research. In the present study, the glucose level which was used as a model for high-hyperglycemic-induced cells, associated with the diabetic condition was in agreement with studies done by Luo et al. [20]. Regarding the results of this study, there was elevated gene expression and ALP activity, which showed an increased proportion of osteoblast cells in the cultures treated with SV which were induced with hyperglycemia under osteoblastic differentiation medium compared to the control group.

Limitations of the study

In vitro culture conditions cannot replicate the active environment that involves the in vivo bone-implant interaction, and their results can only be established in animal models and clinical trials. SV has been commonly used for reducing cholesterol levels; however, more studies are essential in the field of bone regeneration. The ideal dosage, the efficiency for humans, and side effects are to be studied. The cytotoxicity and cell functions revealed by osteosarcoma cells may not be characteristic of those in human primary osteoblasts. However, in vitro models for bone research studies are used with the MG-63 cell line, and the results are recognized which are similar to that of primary human osteoblasts.

## Conclusions

The results of this study demonstrated that the antiresorptive drug namely simvastatin had a significant and direct effect on osteogenesis in osteoblasts in the MG-63 cell line induced with hyperglycemia under osteoblast differentiation medium. Hence this study provides an appreciated step toward a better consideration of treatment options and success anticipated for dental implant therapy in patients with controlled diabetes.
